# Genetic Analysis of Intracapillary Glomerular Lipoprotein Deposits in Aging Mice

**DOI:** 10.1371/journal.pone.0111308

**Published:** 2014-10-29

**Authors:** Gerda A. Noordmans, Yuan Huang, Holly Savage, Marcory C. R. F. van Dijk, Gert Schaart, Marius A. van den Bergh Weerman, Peter Heeringa, Jan-Luuk Hillebrands, Ron Korstanje, Harry van Goor

**Affiliations:** 1 Department of Pathology and Medical Biology, University of Groningen, University Medical Center Groningen, Groningen, The Netherlands; 2 The Jackson Laboratory, Bar Harbor, Maine, United States of America; 3 Department of Pathology, Amsterdam Medical Center, Amsterdam, The Netherlands; 4 Department of Human Movement Sciences, NUTRIM, Maastricht University Medical Centre, Maastricht, The Netherlands; University Medical Center Utrecht, Netherlands

## Abstract

**Background:**

Renal aging is characterized by functional and structural changes like decreased glomerular filtration rate, and glomerular, tubular and interstitial damage. To gain insight in pathways involved in renal aging, we studied aged mouse strains and used genetic analysis to identify genes associated with aging phenotypes.

**Methods:**

Upon morphological screening in kidneys from 20-month-old mice from 26 inbred strains we noted intracapillary PAS-positive deposits. The severity of these deposits was quantified by scoring of a total of 50 glomeruli per section (grade 0–4). Electron microscopy and immunohistochemical staining for apoE, apoB, apoA-IV and perilipin-2 was performed to further characterize the lesions. To identify loci associated with these PAS-positive intracapillary glomerular deposits, we performed haplotype association mapping.

**Results:**

Six out of 26 mouse strains showed glomerular PAS-positive deposits. The severity of these deposits varied: NOD(0.97), NZW(0.41), NON(0.30), B10(0.21), C3 H(0.9) and C57BR(0.7). The intracapillary deposits were strongly positive for apoE and weakly positive for apoB and apoA-IV. Haplotype association mapping showed a strong association with a 30-Kb haplotype block on Chr 1 within the *Esrrg* gene. We investigated 1 Mb on each site of this region, which includes the genes *Spata17, Gpatch2*, *Esrrg*, *Ush2a* and *Kctd3*.

**Conclusions:**

By analyzing 26 aged mouse strains we found that some strains developed an intracapillary PAS and apoE-positive lesion and identified a small haplotype block on Chr 1 within the *Esrrg* gene to be associated with these lipoprotein deposits. The region spanning this haplotype block contains the genes *Spata17, Gpatch2*, *Esrrg*, *Ush2a* and *Kctd3*, which are all highly expressed in the kidney. *Esrrg* might be involved in the evolvement of these glomerular deposits by influencing lipid metabolism and possibly immune reponses.

## Introduction

Aging is characterized by a gradual loss of normally functioning cell mass and requisite cell functions. Age-related decline in renal function and structure predicts shorter lifespan. Physiological aging and/or stress-induced premature senescence of the kidney sets off a process leading to renal cellular senescence and increased risk for chronic kidney disease. Gender, ethnicity and comorbidities play an important role in the rate of renal decline. Although renal aging is considered a physiological phenomenon, it affects organ homeostasis as well as responses to acute and chronic injury. Decreased regeneration capacity makes aging individuals more vulnerable to superimposed stress factors and end-stage renal failure [1], [2]. Focusing on the senescence of the glomerulus there is an increasing number of small sclerotic glomeruli and a compensatory increase in the size of functional glomeruli with age [3]. Understanding age related renal damage may provide tools for preventive and therapeutic means.

Experimental mouse models showed that both sex and genetic background are associated with the progression of renal damage. These phenotypic and genotypic differences are important to define causal factors involved in the progression of damage. Using mice for studying the genetics of aging is ideal since they have a relatively short lifespan and they share 99% of their genes with humans [4], [5]. With large numbers of mouse inbred strains, haplotype association mapping (HAM) can be performed utilizing high-density single-nucleotide polymorphisms (SNP) data from the inbred mice to identify chromosomal haplotypes associated with phenotypic traits of interest [6].

The Nathan Shock Center at The Jackson Laboratory studies genetically diverse inbred mouse strains over time to better characterize aging. Upon screening of the kidneys of these mice we identified the presence of glomerular intracapillary deposits in a minority of strains at old age. In the present study we therefore specifically quantified and characterized these typical glomerular morphological changes in 20-month-old male mice from 26 inbred strains. To localize candidate chromosomal regions associated with these changes we used haplotype association mapping.

## Materials and Methods

### Ethics Statement

All experiments were approved by The Jackson Laboratory’s Animal Care and Use Committee.

### Mice and Tissue Collection

This study is part of a comprehensive aging study carried out by The Nathan Shock Center at The Jackson Laboratory. Age-related phenotypes were measured every six months and made available on the Mouse Phenome Database (www.jax.org/phenome). Kidneys were collected at 6, 12 and 20 months for histopathological and molecular evaluation [7].

Groups of 30 males from different inbred strains were housed in a climate-controlled facility with a 12L:12D light-dark cycle and provided free access to food and water throughout the experiment. After weaning, mice were maintained on a chow diet (Lab diet 5K52, PMI Nutritional International, Bentwood, Mo). Ten males from each strain were sacrificed at 6, 12, and 20 months of age and both kidneys were collected. The left kidney was snap frozen in liquid nitrogen and stored at −80°C while the right kidney was fixed in Bouin’s followed by embedding in paraffin.

### Histological staining and scoring

For morphological evaluation periodic acid-Schiff (PAS) staining was performed on 3 µm paraffin sections. Additional sections were used to exclude deposition of amyloid (Congo Red), fibrin (Martius Scarlet and Blue) or diabetes-related accumulation of non-specific proteins (Jones Methenamine silver stain).

Glomeruli from 20-month-old male mice from 26 inbred strains were screened on a PAS staining. Each animal was evaluated for the presence of glomerular intracapillary deposits. Deposits from the affected mice were evaluated semiquantitative by giving a grade for the presence of deposits on a scale of one to four: 1, <25% of the glomerulus; 2, 25–50%; 3, 51–75%; 4, >75% of the glomerulus contains deposits. A total of 50 glomeruli per section was evaluated.

### Electron microscopy

Electron microscopy was performed to study the lesion at an ultrastructural level. Since kidney tissue was not fixed specifically for electron microscopy, dissected paraffin material was processed for electron microscopy. After fixation in 1% osmiumtetroxide in toluene, the tissue samples were embedded in epoxyresin LX-112. LM sections were stained with toluidine blue. EM sections were stained with tannic acid, uranyl acetate and lead citrate and examined using a Philips CM10 transmission electron microscope (FEI). Images were acquired using a digital transmission EM camera (Morada 10–12, Soft Imaging System, RvC, Soest, NL) using the software Research Assistant (RvC, Soest, NL).

### Immunohistochemistry

Immunohistochemical staining for apoE, B and A-IV was performed on 3 µm paraffin sections to evaluate the accumulation of lipoproteins in the glomerular deposits. Paraffin sections were deparaffinized and antigen retrieval was performed by placing the sections in Tris/HCl buffer at 37°C overnight (apoE and apoA-IV). Following a wash in PBS for 5 minutes, endogenous peroxidase was blocked in PBS, containing 0.03% H_2_O_2_ for 30 minutes at room temperature. After washing for 5 minutes in PBS the sections were incubated in appropriate dilutions of rabbit anti-mouse polyclonal antibodies directed to apoA-IV apoE and apoB [8]. After another wash in PBS, the goat anti-rabbit antibody was applied for 30 minutes at room temperature in a dilution of 1∶100 in 1% BSA/PBS, containing 1% normal mouse serum. After washing with PBS for 5 minutes a rabbit anti-goat antibody was applied. Peroxidase activity was developed in diaminobenzidine + H_2_O_2_ for 10 minutes at room temperature. Counterstaining was performed using haematoxylin.

Immunohistochemical staining for perilipin-2 was performed on 3 µm paraffin sections to evaluate the presence of this protein marker of lipid droplets. After deparaffinization in xylene, sections were rehydrated and thereafter boiled for 5 minutes in 0.01 M Sodium Citrate Buffer, pH 6.0 for antigen unmasking. After cooling down, sections were incubated for 5 minutes in 3% hydrogen peroxide followed by a permeabilization step for 10 min in 0.1%BSA, 1% Triton X-100 in TBS. Sections were blocked for 1 hour at RT with 5% normal goat serum (NGS) in 0.05% Tween 20/TBS. The primary polyclonal guinea pig-antibody directed to PLIN2 (Progen Biotechnik GmbH; Heidelberg; Germany) was diluted 1∶100 in 5% NGS, 0.05% Tween20/TBS and incubated overnight at 4°C. Slides were washed one time for 5 min in 0.05% Tween20/TBS and two times for 5 min in TBS. Secondary antibody incubation for 60 minutes at RT with a horseradish conjugated goat anti-guinea pig antibody (DAKO, Glostrup, Denmark) diluted in 5% NGS, 0.05% Tween20/TBS. After a second wash procedure slides were stained for 10 minutes with DAB (Envision, DAKO), washed for 1 minute with water. Nuclei were counterstained with Mayers Haematoxilin.

### Haplotype Association Mapping

Association mapping for the glomerular intracapillary deposits was performed using the Efficient Mixed Models Association (http://mouse.cs.ucla.edu/emma), which uses a linear mixed model algorithm to control for population structure and genetic relatedness [9]. We used the strain mean as phenotype input and carried out the analysis using a panel of 623,124 SNPs from the Mouse Diversity Genotyping Array, a high-density mouse genotyping array that captures the known genetic variation present in the laboratory mouse [10]. Non-informative SNPs (i.e. SNPs that were not polymorphic between the strains used in our study) were removed from this data set, which resulted in a total of 274,648 informative SNPs. Each SNP was evaluated individually and a P-value was recorded as the strength of the genotype-phenotype association. All P-values were transformed using –log10 (P-value) in the scan plot (score).

Genome sequences within the candidate regions were compared between the different strains based on their haplotype distribution using the Sanger institute Mouse Genomes database.

### Real-time PCR of five associated genes

RNA was isolated from kidney samples using the Trizol method. Samples were diluted and 2 µg was used for cDNA synthesis using the QuantiTect RT kit (Qiagen). mRNA levels were determined in kidney, using a primer set designed by Primerdesign Ltd (Southampton, UK) for *Spata17* (forward: 5′-TTAGCCTACCCTGACCCTTC-3′, reverse: 5′-CAAGCAACTGGA CAGAGA AAT-3′), *Gpatch2* (forward: 5′-TGGATTTGCTGAAACTGGAGAA-3′, reverse: 5′-CGGGTGGTGAACATTGTAGG-3′), *Esrrg* (forward: 5′-GATCTATGCCATGCCTG ACC-3′, reverse: 5-TCCAATGATAACC ACCAACTCTC-3′), *Ush2a* (forward: 5′-CCCTCC AACTGCTGATT TCC-3′, reverse: 5-CTGAAGGTGCTGGCATTGAA-3′), and *Kctd3* (forward: 5′-CAAGAAAAGGTCAGCAGAGGAT-3′, reverse: 5-GAGGATGCCAGATAG GGAACT-3′). RT-PCR was performed using the 7900 HT Sequence Detection System (Applied Biosystems, Inc., Foster City, CA, USA) using SYBR green. mRNA levels were expressed relative to those of the beta-2 microglobulin gene (*B2 m*).

### Statistics

Quantitative data are given as mean ± standard deviation (SD) or as mean ± standard error (SE). The Student *t-test* was used to analyze the expression differences. Linear regression was used to test for correlation. A P-value of less than 0.05 was considered statistically significant.

## Results

### Histology

**Figure 1:**
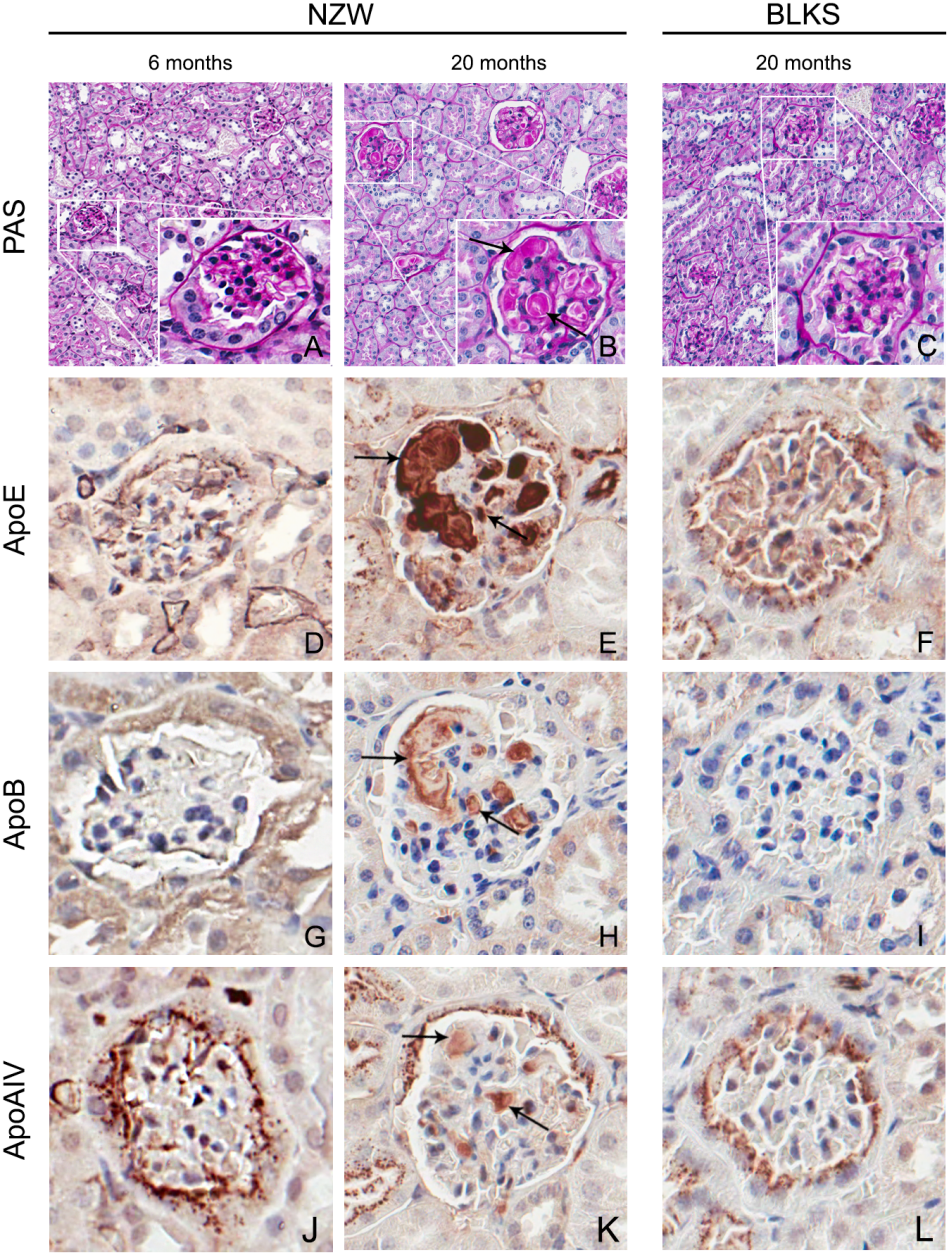
Intracapillary glomerular deposits. Representative example of an unaffected NZW 6-month-old, an affected NZW 20-month-old and an unaffected C57BLKS 20-month-old male mouse using PAS (A–C), apoE (D–F), apoB (G–I), and apoA-IV (J–L) stainings. Deposits are seen in the glomerular capillary lumina (arrows). Immunohistochemistry staining shows that these deposits are strongly positive for apoE (E, arrows) compared to the younger NZW mouse (D) and a mouse of a strain negative for glomerular deposits (C57BLKS) (I). The deposits are weakly positive for apoB (H, arrows) and apoA-IV (K, arrows) compared to the 6-month old NZW mouse (G, J) and C57BLKS control (I, L).

**Figure 2:**
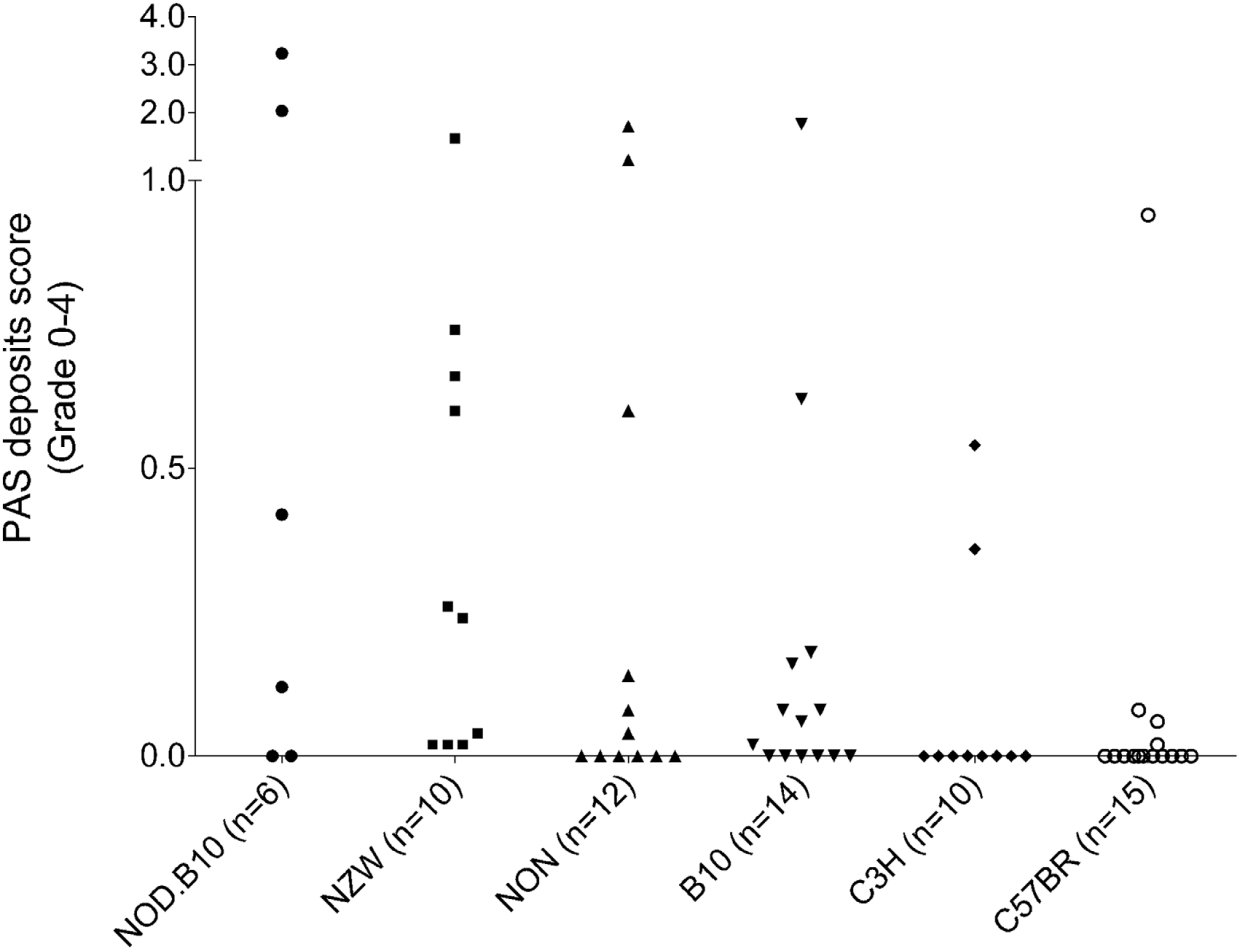
Severity of the deposits. Scoring of the severity of the deposits in 20-month-old male mice of the six affected strains: B10 (n = 14), C57BR (n = 15), NOD.B10 (n = 6), NZW (n = 10), C3 H (n = 10) and NON (n = 12). The score represents the mean percentage of the total of 50 glomeruli affected (grade 0–4).

Morphological evaluation was performed on a PAS staining for all the 20-month-old male mice of 26 inbred strains. We noted a glomerular phenotype in 6 strains (NOD, NZW, NON, B10, C3 H and C57BR), characterized by PAS-positive intracapillary deposits in the glomeruli (Figure 1B). These deposits were negative for Congo Red, MSB and MZ staining, thereby excluding amyloid, fibrin or diabetes related deposits respectively (data not shown). The severity of the deposits varied between the mouse strains: NOD.B10 (mean: 0.97± SD 1.4), NZW (0.41±0.47), NON (0.30±0.55), B10 (0.21±0.48), C3 H (0.09±0.19) and C57BR (0.07±0.24) and the severity between mice within one strain varied as well (Figure 2). The strains that contained deposits at an age of 20 months were also evaluated at 12 months to study the association of these deposits with aging. From the 12-month-old male mice, only NON (0.33±0.6) and NOD (0.08±0.2) showed PAS-positive deposits. At 6 months of age NON showed a glomerular deposit score of mean 0.11±0.2, depicting the age associated increase in glomerular deposits.

### Characterization of the glomerular deposits

**Figure 3:**
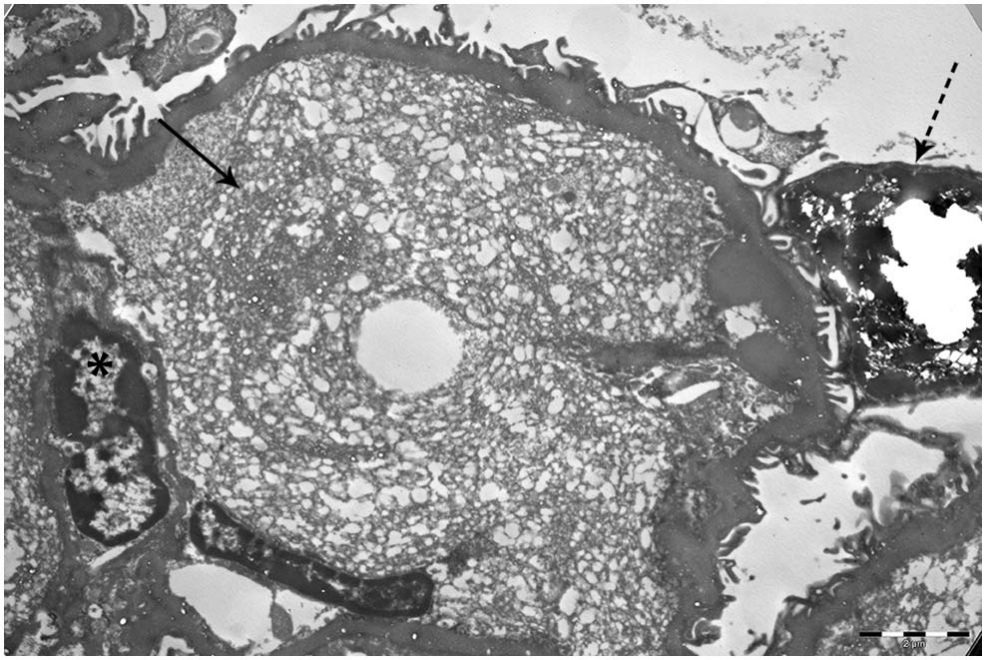
Characterization of the glomerular deposits. Elektron microscopy of an affected glomerulus of a 20-month-old NOD.B10 mouse shows filling of the intracapillary lumina with optically lucent areas, suggestive of lipid vacuoles. Intracapillary lumina (closed arrow), nucleus (*) and podocyte (dashed arrow).

**Figure 4:**
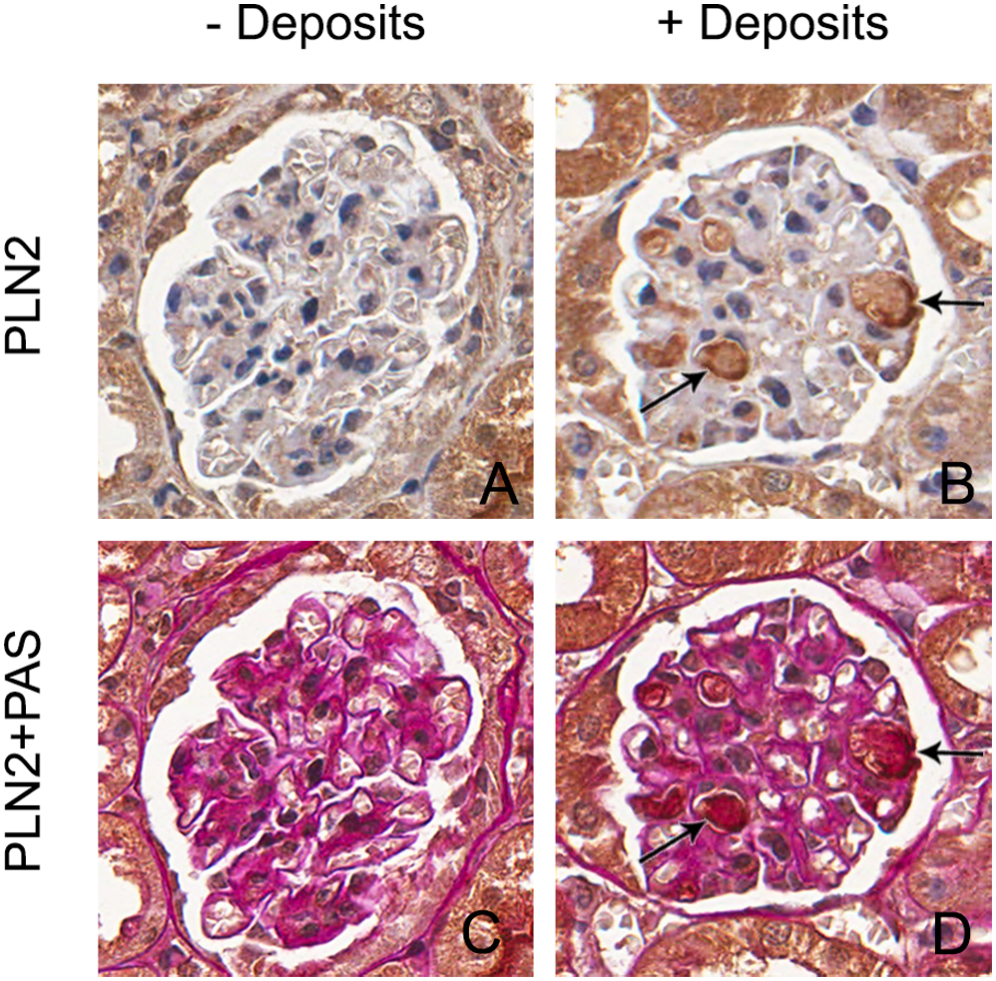
Glomerular deposits express a lipid droplet surface protein. Glomeruli with intracapillary deposits show positive staining for a protein that binds to the surface of lipid droplets, perilipin-2 (B, D) in contrast to glomeruli without deposits, which are negative (A, C).

Electron microscopy of a 20-month-old mouse with glomerular deposits shows the presence of optically lucent areas in the capillary lumina suggestive of lipid vacuoles (Figure 3). Thickening of the basement membrane, characteristic for diabetes, was not seen. Immunohistochemical staining showed that the intracapillary deposits were strongly positive for apoE (mainly LDL, VLDL) (Figure 1E) and weakly positive for apoB (mainly LDL, VLDL) (Figure 1 H) and apoA-IV (HDL) (Figure 1 K). Frozen material was not available from these mice, therefore an Oil red O staining for neutral lipids could not be performed. However, the perilipin-2 staining, which is a protein coating lipid droplets, shows positive staining of the glomerular deposits (Figure 4).

### Haplotype association mapping

**Figure 5:**
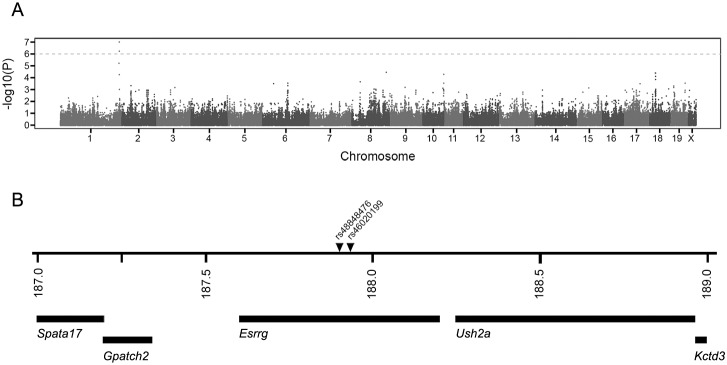
Genome wide HAM results. Genome-wide scan for intracapillary glomerular deposits. A peak reaching the significant threshold of *P*<10^−6^ is seen on Chr 1 (A). The haplotype block of six SNPs between rs48848476 and rs46020199 is associated with glomerular deposits. The region spanning 1 Mb on each site from this area includes the genes *Spata17, Gpatch2, Esrrg, Ush2a,* and *Kctd3* (B).

A panel of 623,124 SNPs was used for the haplotype association mapping. After removing the non-informative SNPs, analysis was carried out with a total of 274,648 SNPs. For each SNP the association between genotype and the presence or absence of the glomerular intracapillary deposit phenotype was analyzed individually and a P-value was recorded as the strength of the genotype-phenotype association. We focused on associations with a P-value of less than 10^−6^. Figure 5A summarizes the genome-wide HAM results for glomerular intracapillary deposits in males at 20 months of age. The only peak above the threshold of P<10^−6^ was seen on chromosome 1. The analysis identified a small 30-Kb haplotype block of six SNPs (between rs48848476 and rs46020199) within the *Esrrg* gene with a P-value of 9.59×10^−8^, which is different between strains with glomerular deposits and without glomerular deposits. The HAM analysis data have been submitted to the Mouse Phenotype Database at The Jackson Laboratory (http://phenome.jax.org/). To be conservative, we investigated the region spanning 1 Mb on each site of this region. According to the Ensembl Genome Browser (NCBI m37 assembly), Spermatogenesis associated 17 (*Spata17*), G patch domain containing 2 (*Gpatch2*), Estrogen related receptor gamma (*Esrrg*), Usher syndrome 2a (*Ush2a*), and Potassium channel tetramerisation domain containing 3 (*Kctd3*) are found within this 2 Mb region (Figure 5B). Comparing genome sequences using the Sanger Institute Mouse Genome database showed no sequence differences in the coding regions of these genes between the strains with and without glomerular deposits (www.sanger.ac.uk/resources/mouse/genomes/).

### Expression of associated genes

**Figure 6:**
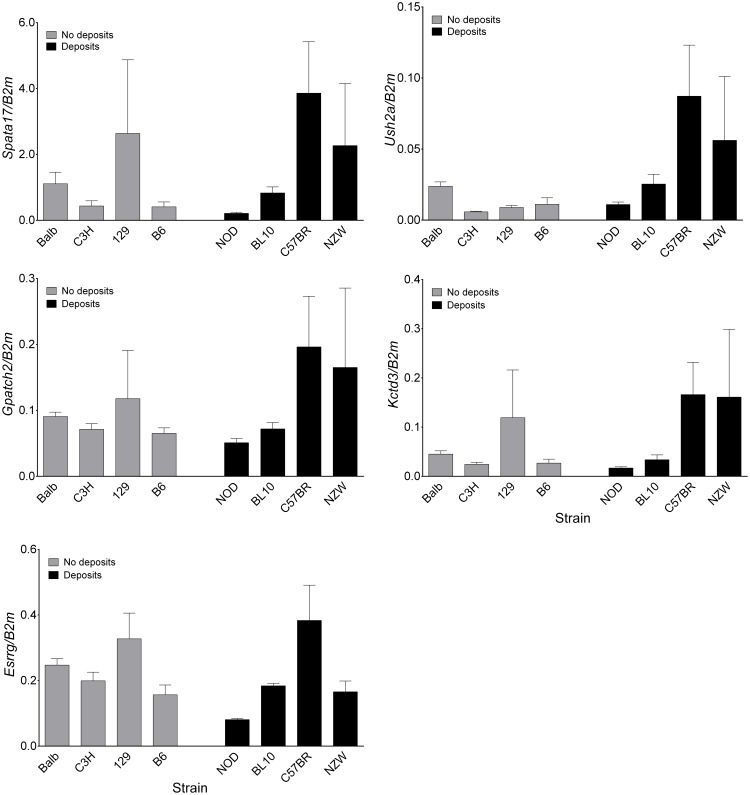
Expression levels of the five associated genes. Rt-PCR for *Spata17, Gpatch2, Esrrg, Ush2a,* and *Kctd3* (n = 3–5). The results represent the mean ± SE. Comparing all animals (regardless of strain) with and without deposits using a t-test showed no significant difference for any of the genes.

Performing real-time PCR for *Spata17, Gpatch2*, *Esrrg*, *Ush2a* and *Kctd3* on kidney RNA from several strains with and without glomerular deposits showed a high expression of all genes in the kidney, but no significant difference in expression levels between strains with and without deposits (Figure 6).

## Discussion

In the present study we found intracapillary glomerular deposits in 6 out of the 26 mouse strains at 20 months of age. These deposits are age-associated since they were absent or less extensive in mice of 12 and 6 months of age. EM and immunohistochemistry indicated that the deposits contain lipoproteins, suggestive of LDL and VLDL accumulation. Using genetic analysis, this study identified a small 30-Kb haplotype block on Chr 1 of six SNPs within the *Esrrg* gene that is associated with the age-related lipoprotein deposits. To be conservative, we investigated the region spanning 1 Mb on each site of this region, which includes the genes *Spata17, Gpatch2*, *Esrrg*, *Ush2a* and *Kctd3*.

The glomerular deposits seen in our mice are in the intracapillary lumina of the glomerulus. Several renal glomerular diseases show abnormal lipid depositions, the human disorder lipoprotein glomerulopathy (LPG) also shows intracapillary glomerular deposits. LPG is a rare disease typically seen in patients in East Asia, but also in Caucasians in Europe and the United Sates. LPG is characterized by proteinuria, occlusion of glomerular capillaries by lipoprotein-containing material and elevated serum apolipoprotein E levels. If left untreated LPG progresses to end-stage renal disease [11]–[14]. Recently a study into patients with the apoE Kyoto mutation was published in which fenofibrate treatment could induce remission of the disease. The existence of asymptomatic carriers suggested that other genetic and epigenetic factors are involved in the pathogenesis of LPG [15]. In our affected mouse strains, we showed that de deposits contain lipoproteins. However, by searching the phenome database (www.jax.org/phenome) we could not find a consistent increase in ACR or non-HDL levels for the affected strains compared to the strains without deposits. Diabetic nephropathy with nodular glomerular lesions was excluded since we did not observe expansion of the mesangium on the PAS staining or thickening of the glomerular basement membrane by EM and the deposits did not show a specific Jones MZ stain for diabetes.

In line with our results, Watanabe et al describe glomerular deposits in a strain of 3 months old NON mice similar to the deposits seen in our aged mice [16]. However, lymphoid follicle-like structures around renal arterioles and tubular dilatation were found in that particular study and absent in our affected strains.

Other mouse models in which lipoprotein glomerulopathies are described include virus-mediated transduction of the apoE Sendai mutation in apoE-deficient mice and aged apoE-deficient mice. A contrasting study claims that the latter mice do not develop histological changes identical to those seen in human lipoprotein glomerulopathy [17]–[19]. Another mouse model with NON and NOD.B10 mice was performed to distinguish gene expression changes due to diabetes compared to changes due to proteinuria. Interestingly, in this study NON mice presented with lipoprotein glomerulopathy. Performing a micro-array analysis, up-regulation of genes involved in lipid processing and immune related response were found between the NON mice with deposits and without deposits [20]. Sustained inflammation as a predisposing factor for lipoprotein glomerulopathy was proposed in chronic autoimmune GVHD in FcRγ-deficient mice [21]. Although several glomerular deposit lesions are thought to resemble human LPG, the morphological difference can be made by performing EM and lipid stains [19], [22]. Since the kidneys of our cohort were not ideally fixated for EM analysis and lipid stainings it is hard to elucidate if these lipoprotein deposits are similar to those seen in human LPG. However, more interesting in our study is the possible etiologic role of apoE. The lipids and apoE abnormalities seen in our affected mouse strains are increasing with age. Lipids are known to play a role in glomerulosclerosis [8], [23], [24] and apoE abnormalities seem to have a genetic or epigenetic factor in several renal diseases[25]–[28].

To find the genomic location associated with this phenotype we performed haplotype association mapping (HAM). HAM can be used to find an association between haplotype blocks and a specific phenotype by using large numbers of mouse inbred strains. Previously published data on the currently used cohort reports on genes involved in the age-related susceptibility for albuminuria and mesangial matrix expansion and indicated *Nalcn* as an important player in osmoregulation [29]–[31]. Performing haplotype association mapping we found a significant association between the intracapillary glomerular deposits and a 30-Kb haplotype block on Chr 1 within the *Esrrg* gene. The region spanning 1 Mb on each side of this haplotype block contains the genes *Spata17, Gpatch2*, *Esrrg*, *Ush2a* and *Kctd3.* Estrogen-related receptor gamma (*Esrrg*) is expressed in tissues with high metabolic activity like the heart and kidney and coupled to circadian clocks and metabolism [32], [33]. It is considered an orphan nuclear receptor since an endogenous ligand is still unknown. *Esrrg* KO mice have cardiomyopathy, a cardiac conduction defect and kidney and urinary tract anomalies and die perinatally [34], [35]. This nuclear receptor is an important regulator of mitochondrial biogenesis and lipid transport and metabolism [36], [37]. Hepatic gene expression of *Esrrg* was upregulated 2.4-fold in a study on candidate genes that affect lipid and lipoprotein metabolism in response to fenofibrate treatment, interestingly the same treatment induced remission of lipoprotein glomerulopathy in patients with the apoE Kyoto mutation [15], [37].

In an NZW-derived strain, similar loci (i.e. on Chr1 and *Esrrg*) were found to be associated with cGVHD susceptibility and increased CD4^+^ T cell intrinsic activation by decreased mitochondrial mass and alteration of mitochondrial function [38]. As a regulator of metabolism, *Esrrg* was proposed as an important player in regulating immune functions and autoimmunity.

Many of the former studies on glomerular deposits in mice suggest a role of the immune system in causing the evolvement of deposits. Since aging is known to be associated with changes in the immune response, the glomerular deposits might be caused by age-related immune system changes, managed through metabolic changes by *Esrrg*.

The role of the other four associated genes in kidney function or lipid metabolism is unknown. KCTD3 is an uncharacterized member of the potassium channel tetramerization-domain containing protein family, widely expressed in brain, liver, heart and kidney. Recently it was shown that KCTD3 interacts with hyperpolarization-activated cyclic nucleotide-gated channel subunit 3 (HCN3) in mouse brain [39]. HCN3 is also expressed in the kidney, suggesting that KCTD3-HCN3 complexes may exist in the kidney [40]. It is also likely that KCTD3 function may extend beyond the regulation of HCN3. G patch domain containing 2 (*Gpatch2*) was shown to be overexpressed in a great majority of breast cancer cases, probably regulating a broad range of cellular functions [41]. Spermatogenesis associated 17 (*Spata17*) is a testis expressed gene involved in spermatogenesis and the gene USH2A encodes for usherin, a basement membrane protein. USH2A mutation can cause Usher syndrome that results in hearing loss and retinitis pigmentosa.

In our study, we did not find a coding difference or a difference in expression of *Spata17, Gpatch2*, *Esrrg*, *Ush2a* and *Kctd3* between strains with and without glomerular deposits.

A possible explanation for that is that we used whole kidney tissue, while the differences might only be present in glomeruli or circulating T cells. Since there is a large variation between the strains positive for glomerular deposits, we looked at each strain individually showing a borderline significant negative correlation between the level of *Esrrg* and the number of deposits (P = 0.07) (data not shown). This indicates that a reduced *Esrrg* expression increases the deposit formation. Furthermore, from overexpression experiments in cell lines it is known that e.g. *Esrrg* message and protein levels are not tightly correlated, suggesting an important role for posttranscriptional regulation [38]. Therefore, it might be possible to find a difference in expression of target genes of *Esrrg*, very likely using different cofactors to dictate its function [36], [42]–[44].

In conclusion, we describe intracapillary glomerular deposits strongly positive for apoE in several aged male mouse strains. Performing haplotype association mapping we revealed a significant association between this phenotype and a 30-Kb haplotype block on Chr 1 within the *Esrrg* gene. The 2 Mb region spanning this haplotype block includes the genes *Spata17, Gpatch2*, *Esrrg*, *Ush2a* and *Kctd3.* Although we did not find a coding or expression difference between the strains, Estrogen-related receptor gamma is known to be involved in lipid transport and metabolism, and possibly in regulating immune functions. The exact mechanism of the evolvement of glomerular intracapillary deposits in aged mice needs to be elucidated.
